# Effects of Grape Seed Extract Supplementation on Endothelial Function and Endurance Performance in Basketball Players

**DOI:** 10.3390/ijerph192114223

**Published:** 2022-10-31

**Authors:** Hosung Nho, Kyung-Ae Kim

**Affiliations:** 1 Department of Liberal Arts and Science, Suwon Women’s University, Onjeong Street 72, Gweonseon-gu, Suwon-si 16632, Korea; 2 Human IT Solution, Gogum Building 1F, 2F, Banpo-daero, 55, Seocho-gu, Seoul 06670, Korea

**Keywords:** grape seed extract, oxygen consumption, endurance performance, endothelial function, basketball players

## Abstract

While dietary polyphenols supplements can improve endothelial function and blood flow to exercise, the effects of chronic supplementation with grape seed extract (GSE) containing a high dose of polyphenols on endurance performance are not known. Accordingly, in 12 elite athletes, we compared the effects of both GSE and placebo (PL) on submaximal VO_2_, time to exhaustion performance, and endothelial function during progressive cycling exercise for 14 days. Endothelial function was evaluated from the brachial artery via flow-mediated dilation (FMD). Compared to PL, GSE decreased submaximal VO_2_ at 80% and 120% of VO_2peak_ and increased the time to exhaustion (*p* < 0.05). GSE also resulted in FMD-induced increase in brachial artery diameter (14.4 ± 5.2% vs. 17.6 ± 4.5%, *p* = 0.035). We demonstrated that chronic supplementation with GSE improved endurance performance and these effects may partially be due to vasodilation in active skeletal muscle mediated by enhanced endothelial function. Thus, our results suggest that GSE appears to be an ergogenic nutraceutical that can improve exercise performance in elite athletes.

## 1. Introduction

Dietary supplementation to improve exercise and athletic performance has received widespread attention in sports. These supplements referred to as ergogenic aids have been known to enhance strength or endurance, exercise efficiency, recovery from exercise, and increase exercise tolerance to high-intensity exercise [1,2,3,4,5]. Proper supplementation strategies can help elite athletes endure more intensive training and competition. Accordingly, in the sports field, many athletes increasingly rely on dietary supplements, believing that they can promote recovery between training sessions and enhance competitive performance (e.g., cycling, running, rowing, and triathlon) [6,7].

When endurance athletes participate in a repetitive and high-intensity competition, their performance is limited by various physiological factors such as muscle fatigue, energy efficiency, and maximum oxygen consumption [8]. For the past several years, dietary nitrate (NO_3_^−^) supplementation with beetroot juice has become a popular nutraceutical among athletes due to an improvement in blood flow, oxygen efficiency, and exercise tolerance [2,3,9]. The main production of nitrate metabolism is nitric oxide (NO) which has a major role in blood pressure and vascular smooth muscle relaxation. NO contributes to an increase in organ blood flow [10]. NO is endogenously produced by the oxidation of L-arginine through NO synthases [11] and exogenously from dietary nitrate via the NO_3_^−^-NO_2_^−^ -NO pathway [12].

Using nutritional polyphenols supplements is of considerable interest to elite athletes for improving sports performance as well as use in human health. Grapes have been known to be rich in polyphenols which are found mostly in the seeds. Grape seed extract (GSE) primarily accounts for flavanols (flavan-3-ol derivatives) that are present in both monomeric and polymeric forms and are the principal active compounds [13]. These active compounds contain monomers of (+)-catechin, (−)-epicatechin, and their gallic acid esters [13]. GSE acts as an antioxidant and has been known to be effective in counteracting exercise-induced oxidative stress [14] which has been known to induce endothelial dysfunction and limit blood flow to exercising muscles [15,16]

Recently, an extract of grape seed has been developed via hydrolysis of specific enzymes [17] that is highly purified and rich in polyphenolic compounds. It has been demonstrated that this extract lowers blood pressure (BP) in human subjects [17,18]. The mechanism underlying the antihypertensive effects of this extract involves the activation of endothelial nitric oxide synthase (eNOS) and in turn production of nitric oxide NO via the improved function of the endothelium [19,20]. NO is generally known to play an important role in increasing blood flow to active skeletal muscles. However, the potential beneficial effects of this highly purified form of GSE on endurance exercise performance have not been examined.

The sport of basketball demands specific skills that are able to be performed under dynamic conditions. For success in basketball performance, players are required to possess high strength, power, and agility. Accordingly, coaches often take less attention to the contribution of the aerobic capacity to improve sports performance in basketball; however, endurance is related to the successful performance of high-intensity work for a longer period of time before the onset of fatigue [21].

Research related to the knowledge of dietary GSE supplementation is not known in elite athletes. Thus, this study hypothesized that chronic dietary GSE supplementation reduces oxygen levels during submaximal exercise and increases time to exhaustion performance due to an improvement in endothelial function in elite athletes.

## 2. Methods

### 2.1. Participants

This study was a randomized double-blind placebo-controlled experimental study that involved 12 division I collegiate basketball players from Kyung Hee University. The Exclusion criteria for this study involved: renal, or cardiovascular disorders, those who consumed antibiotics or supplemental vitamins and minerals, and those who consumed drugs that impact metabolic profiles. Prior to testing, all subjects gave written informed consent. The body composition, including height, weight, body mass index, fat mass, and percent body fat was measured using a body composition analyzer (MC190-EM; Tanita, Tokyo, Japan) (Table 1). All procedures were reviewed and approved by the Kyung Hee University Institutional Review Board (KHU 2014-G21).

### 2.2. Exercise Test Protocol

Resting BP was measured while the participant was in a seated position. At least two measurements were obtained, 2 min. apart, using a sphygmomanometer and pressure cuff. To determine the relative exercise intensities for the three workloads used in this study, a maximal exercise test was conducted using a cycle ergometer. Pulmonary gases were measured on a breath-by-breath basis using an Ultima CPX Metabolic Cart (Medgraphic, St. Paul, MN, USA). The incremental exercise protocol started with 2 min. of unloaded baseline cycling followed by increases in the workload of 30 Watts per minute until they could no longer maintain a pedal cadence of 60 rpm. Rating of perceived exertion (RPE) and heart rate were measured continuously and recorded every minute.

**Figure 1:**
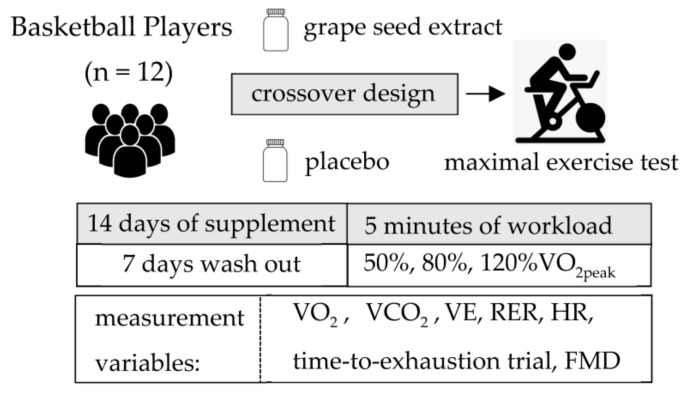
Study design.

All participants then completed two bouts of cycling exercise at constant submaximal workloads corresponding to 50% and 80% of their predetermined VO_2peak_ on the same day. The duration of each workload was 5 min. To avoid muscle fatigue, they completed a supramaximal bout of cycling exercise corresponding to 120% of VO_2peak_ on a different day. During the time-to-exhaustion trial, participants were instructed to cycle for as long as possible at their targeted 60 rpm. The trial is commonly used to assess endurance performance [22]. There was a cessation of exercise when a given workload was not maintained. All participants exercised at the same workloads after PL and GSE supplementation (Figure 1).

### 2.3. Supplementation

Following twenty-four hours after the VO_2peak_ exercise test, a randomized double-blind crossover study design was initiated to determine the effects of 14 days of GSE supplementation (administrated as a single dose of 300 mg in capsule form; MegaNaturalbβ^®^-BP, Polyphenolics Inc., Madera, CA, USA) compared with PL treatment (300 mg of starch). This GSE product is a highly concentrated purified source of polyphenolic flavan-3-ols. The total phenol content determined by the Folin–Ciocalteu method was 95.3% (grams gallic acid equivalent/100 g), contained 2.8% gallic acid, 5.1% catechin plus epicatechin, and 87.4% total oligomers as determined by high-performance liquid chromatography. It was manufactured via hydrolysis using specific proprietary enzymes [17] to produce a product rich in polyphenolic compounds. All participants were instructed to take the GSE around the same time every morning. Neither GSE nor PL supplements were taken for 24 h prior to exercise testing. A one-week washout period separated the supplementation period. Participants were asked to maintain their normal daily activities and food intake during the entire study period. They received a list of vegetables that influenced the production of NO and were asked to record it in a food diary during the duration of the study. When the participants made any major changes, their data were excluded from the data analysis. The investigator administering the exercise tests was blinded to the type of supplementation being consumed by the participants. The main researcher monitored the participants via text message to remind them to follow these guidelines.

### 2.4. Flow-Mediated Dilation (FMD)

The FMD technique was used to determine endothelial function in the brachial artery in the morning after each supplementation. This non-invasive technique is a well-known bioassay of peripheral endothelial function that involves the release of a temporary occlusion of the arm vasculature to induce an acute increase in shear stress [23]. Brachial artery diameter and velocity were measured by an ultrasound 12-MHz linear-array vascular probe (ClearVue 550, Philips, Cambridge, MA, USA). The probe was placed 3–5 cm proximal to the bifurcation of the antecubital fossa. When the images were obtained, the width of the artery was insonated at an angle of 60°. Blood velocity was acquired simultaneously using a pulsed-wave Doppler. The images were obtained and analyzed by the same examiner in a blinded manner. Ten cardiac cycles were evaluated to calculate baseline arterial diameter. To produce reactive hyperemia, the pressure cuff was placed on the upper arm and inflated by 200 mmHg for 5 min followed by rapid deflation. The brachial artery was imaged and recorded for 2 min. The peak diameter was determined as the average of five cardiac cycles. Brachial artery images were selected when they occurred near the end of diastole. The absolute change in diameter was determined, and FMD was expressed as a percent change in diameter from baseline (%FMD).

### 2.5. Statistical Analysis

Statistical analyses were performed with SPSS 21.0 software (SPSS Inc., Chicago, IL, USA) and SAS 9.4 software (SAS Institute Inc., Cary, NC, USA). All variables assessed by the metabolic cart were averaged over the last 30-s periods during a steady-state submaximal exercise. The peak VO_2_ was measured during the time to exertion trial. The total time from beginning to exhaustion was measured. To compare the effects of GSE between groups, a paired *t*-test was used. Statistical significance was accepted at *p* < 0.05. A power analysis (power = 0.80) revealed the necessity for 12 participants to achieve statistically significant results of VO_2_ between PL and GSE supplementations.

## 3. Results

**Figure 2:**
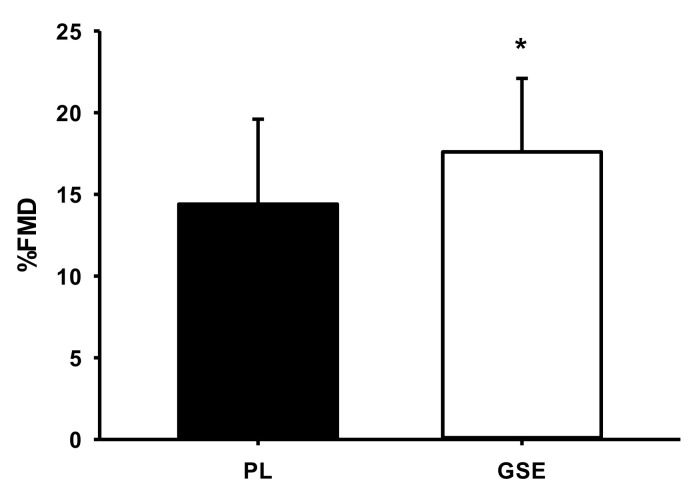
Flow-mediated dilatation after PL and GSE supplementation. FMD: flow-mediated dilation. Values are expressed as means ± SE. * *p* < 0.05. vs. PL supplementation.

Table 2 shows maximum oxygen consumption after PL and GSE Supplementation. There was no difference in VO_2peak_ between GSE and PL supplementation. Table 3, Table 4 and Table 5 illustrate cardiorespiratory responses during submaximal and supramaximal exercise after PL and GSE supplementation. There was no difference in VO_2_ at 50% workload between the two groups, but VO_2_ significantly decreased at 80% and 120% workloads after GSE supplementation compared to PL (*p* = 0.041 and *p* = 0.031, respectively). The time to exhaustion trial had a significant increase after GSE (*p* = 0.035). There were no significant differences in the other variables across workloads. Figure 2 shows flow-mediated dilatation after PL and GSE supplementation. FMD was significantly increased after GSE compared to PL (22%, *p* = 0.035).

## 4. Discussion

To my knowledge, this study was to investigate the effects of chronic supplementation with GSE on VO_2_, endurance performance, and endothelial function in division 1 collegiate basketball athletes. GSE substantially reduced the O_2_ cost of cycling at a fixed submaximal work rate and the time to exhaustion trial was significantly increased during supramaximal exercise compared to PL. Furthermore, endothelial function was improved after GSE. Our study demonstrated that an improvement in endurance performance was likely due to an increase in NO bioavailability.

The ergogenic effect of GSE may be mediated by the effect of NO in the improvement of mitochondrial oxygen efficiency. It has been reported that increasing NO levels after the beetroot juice (BRJ) supplementation with NO donors induced benefits in exercise performance because the supplement can decrease the cost of ATP production which enhances mitochondrial ATP production efficiency [2,24]. Although the mechanism utilized by the nitrates found in vegetables such as beetroot, lettuce, and spinach to improve the efficiency of submaximal exercise is still unclear, it has been known to be due to an enhanced oxidative phosphorylation efficiency as assessed by the amount of oxygen consumption per ATP production (P/O ratio) [25]. A previous study demonstrated that the increase in mitochondrial P/O ratio was highly correlated with the reduction in oxygen cost during exercise after nitrate supplementation [25]. Thus, a reduction in submaximal oxygen consumption appears to be a good index of an improvement in mitochondrial efficiency. To demonstrate this hypothesis, Bailey et al. and others investigated the effects of BRJ supplementation in a time trial performance in humans and found a significant improvement compared to PL [3,26,27]. These findings were attributed to a significant increase in the level of nitrite (NO_2_^−^) following the supplement. This study suggested that dietary nitrate can be an ergogenic aid that can improve sports performance. Our study also found that GSE supplementation lowered VO_2_ during 80% and 120% of VO_2peak_ workloads and increased the time to exhaustion performance compared to PL. Thus, exercise can be likely performed at high intensity for a longer period of time before the onset of fatigue with the same energy production following dietary supplementation with GSE.

The endothelium regulates vascular tone, mainly by stimulating NO production in response to various stimuli [28]. NO is a potent vasodilator that mediates the control of vascular function and blood flow during exercise [29,30]. Despite the fact that dietary nitrate supplementation can act as an ergogenic nutraceutical to improve exercise performance [30,31], high intakes of dietary nitrate may cause harmful effects on health (i.e., cancer). In this regard, there should be some regulations on nitrate concentration in food to minimize safety issues. Considering another alternative source, a recent study investigated the effect of acute GSE supplementation on peripheral vasoconstriction and endothelial function in prehypertensive men [19]. It demonstrated that GSE improved endothelial function and increased peripheral vasodilation. Another study showed that dietary nitrate supplementation increased the production of NO and markedly enhanced FMD, indicating that endothelial function was profoundly improved in college students [30], Similarly, this current study found that endurance performance enhancement, following GSE supplementation, was attributed to improved endothelial function (i.e., increased FMD) and oxygen delivery as a result of increased vasodilation in contracting skeletal muscle. These findings suggest that GSE supplements can increase blood flow to skeletal muscles due to an improvement in NO bioavailability and exercise performance before the development of muscle fatigue.

Our study was limited by not measuring the indices of NO bioavailability (e.g., nitrates and nitrite concentrations). Assessment of these substances would have confirmed our conclusions concerning the contribution of NO bioavailability to GSE-induced improvements in endothelial function and endurance performance. Secondly, each of the pre-treatment conditions was not measured to compare to those of post-treatments. Therefore, our results need to be carefully considered with these facts since the effects of PL or GSE supplementations on endothelial function cardiorespiratory responses were not quantified. This is an important distinction because the change from pre-treatment to post-treatment is necessary to reveal physiological effects.

## 5. Conclusions

In conclusion, although some studies demonstrated that dietary supplementation with NO donors is effective to improve exercise performance, others found no consistent effects. In our study, dietary supplementation with GSE enhanced exercise performance and endothelial function in collegiate basketball players. In consequence, the onset of muscle fatigue may be delayed in athletes performing exercise for longer periods of time. Findings suggest that a small dosage of GSE supplementation can act as an ergogenic aid capable of enhancing O_2_ delivery and endurance performance. Due to the findings from this study, future research should be examined where dietary GSE supplementation improves endurance performance (i.e., time trial performance).

## Figures and Tables

**Table 1:** Physical characteristics of subjects.

Variables	Subjects (n = 12)
Age (years)	20.2 ± 1.0
Height (cm)	190.6 ± 0.1
Weight (kg)	81.5 ± 10.2
BMI (kg/m^2^)	22.9 ± 1.8
Fat mass (kg)	13.1 ± 4.1
Body fat (%)	13.0 ± 2.2
SBP (mmHg)	120.6 ± 12.0
DBP (mmHg)	75.3 ± 6.1
Resting HR (bpm)	62.6 ± 11.2

BMI, body mass index; SBP, systolic blood pressure; DBP, diastolic blood pressure; HR, heart rate.

**Table 2:** Maximum oxygen consumption after PL and GSE supplementation.

Variables	PL Supplementation	GSE Supplementation	*p*
VO_2peak_ (mL/kg/min)	55.6 ± 3.0	55.0 ± 5.8	0.338

PL: placebo, GSE: grape seed extract. Values are expressed as means ± SE.

**Table 3:** Cardiorespiratory responses during the submaximal exercise of 50% of VO_2peak_ after PL and GSE supplementation.

Variables	PL Supplementation	GSE Supplementation	*p*
HR (bpm)	114.5 ± 8.4	114.3 ± 7.2	0.928
VO_2_ (mL/kg/min)	28.4 ± 4.6	27.4 ± 4.1	0.176
VO_2_ (L/min)	2.3 ± 0.4	2.2 ± 0.4	0.163
VCO_2_ (mL/min)	2198.1 ± 400.3	2126.8 ± 313.1	0.306
RR (br/min)	31.8 ± 5.3	31.0 ± 6.6	0.355
Vt (mL)	1712.0 ± 194.6	1712.9 ± 284.3	0.179
VE (L/min)	54.1 ± 9.9	51.9 ± 7.9	0.061
RER	1.0 ± 0.1	1.0 ± 0.1	0.704

HR, heart rate; RR, respiratory rate; Vt, tidal volume; VE, minute ventilation; RER, respiratory exchange ratio. PL: placebo, GSE: grape seed extract. Values are expressed as means ± SE.

**Table 4:** Cardiorespiratory responses during the submaximal exercise of 80% of VO_2peak_ after PL and GSE supplementation.

Variables	PL Supplementation	GSE Supplementation	*p*
HR (bpm)	153.1 ± 8.8	153.7 ± 5.6	0.834
VO_2_ (mL/kg/min)	45.7 ± 4.9	44.3 ± 6.1 *	0.041
VO_2_ (L/min)	3.7 ± 0.4	3.6 ± 0.5	0.041
VCO_2_ (mL/min)	3723.7 ± 519.1	3637.9 ± 471.3	0.566
RR (br/min)	43.1 ± 4.3	44.2 ± 6.5	0.590
Vt (mL)	2209.4 ± 246.8	2132.4 ± 327.4	0.061
VE (L/min)	94.8 ± 12.4	93.0 ± 15.0	0.117
RER	1.0 ± 0.0	1.0 ± 0.1	0.920

HR, heart rate; RR, respiratory rate; Vt, tidal volume; VE, minute ventilation; RER, respiratory exchange ratio. PL: placebo, GSE: grape seed extract. Values are expressed as means ± SE. * *p* < 0.05. vs. PL supplementation.

**Table 5:** Cardiorespiratory responses during the submaximal exercise of 120% of VO_2peak_ after PL and GSE supplementation.

Variables	PL Supplementation	GSE Supplementation	*p*
HR (bpm)	164.2 ± 14.2	167.1 ± 10.4	0.735
VO_2_ (mL/kg/min)	52.4 ± 7.7	50.9 ± 6.4 *	0.030
VO_2_ (L/min)	4.2 ± 0.6	4.1 ± 0.5	0.030
VCO_2_ (mL/min)	4932.4 ± 927.2	4732.2 ± 677.3	0.695
RR (br/min)	58.4 ± 5.8	57.1 ± 6.7	0.728
Vt (mL)	2394.2 ± 507.7	2340.8 ± 445.4	0.756
VE (L/min)	139.1 ± 26.5	130.8 ± 17.3	0.461
RER	1.2 ± 0.1	1.2 ± 0.1	0.885
Time (sec)	128.9 ± 53.0	134.4 ± 58.4 *	0.050

HR, heart rate; RR, respiratory rate; Vt, tidal volume; VE, minute ventilation; RER, respiratory exchange ratio. Values are expressed as means ± SE. * *p* < 0.05. vs. PL supplementation.

## Data Availability

Not applicable.
